# Supplementary material for: NbMLP43 Ubiquitination and Proteasomal Degradation via the Light Responsive Factor NbBBX24 to Promote Viral Infection
Source: Cells. 2023 Feb 11;12(4):590. doi: 10.3390/cells12040590 (PMC9954743; doi:10.3390/cells12040590)
Supplement: Supplementary file 1 [file cells-12-00590-s001.zip › Supplemental figure.pdf]

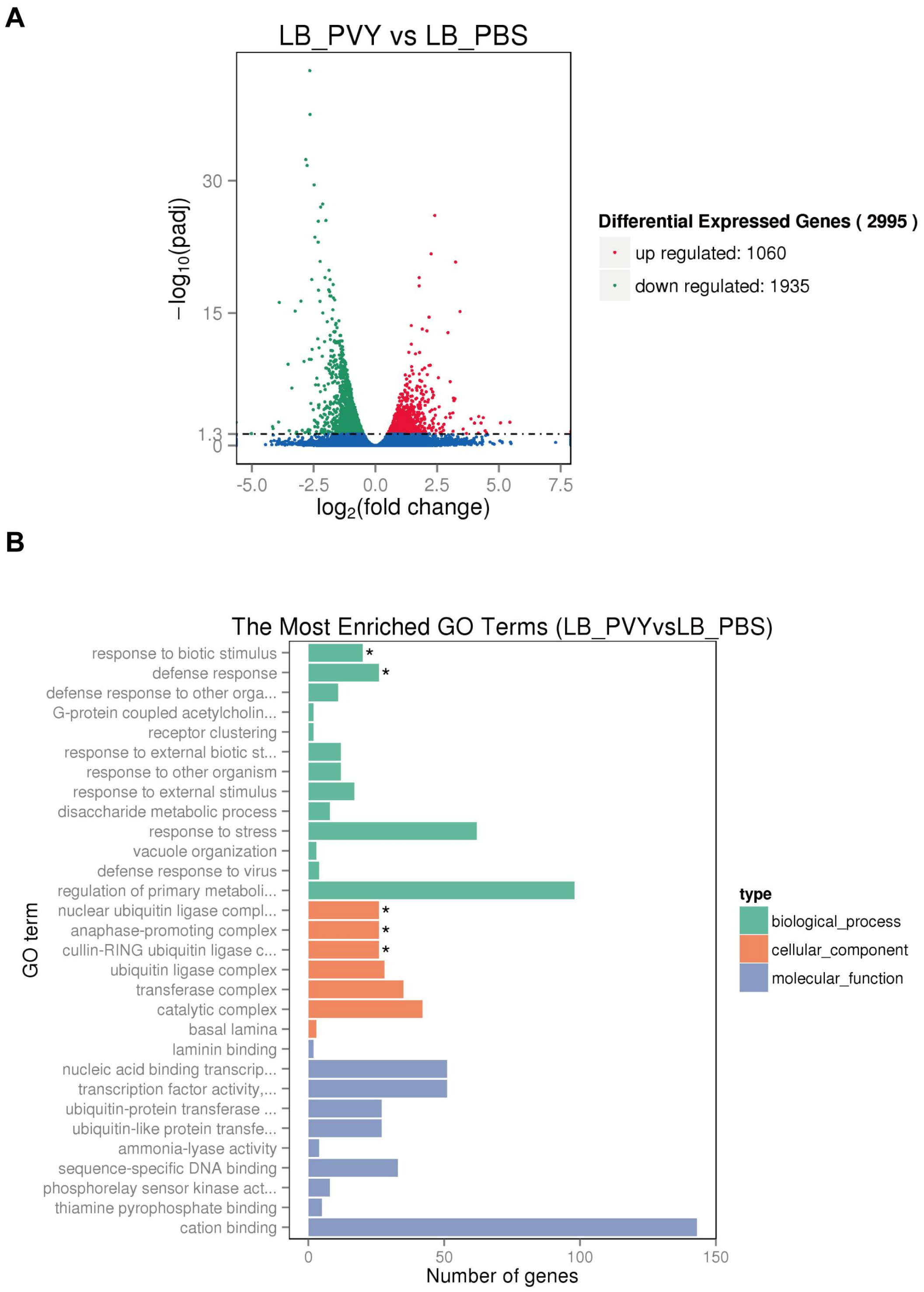

Fig. S1 Identification of MLP43 based on omics data. (A) Differentially expressed genes after PVY infection. (B) The most enriched GO terms of up-regulated genes.

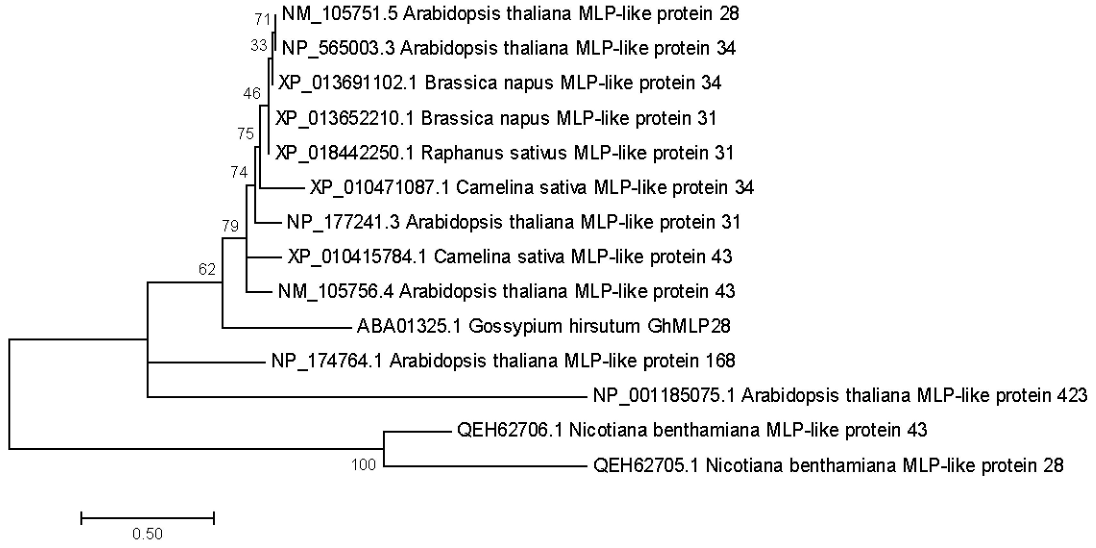

Fig. S2 Phylogenetic tree of NbMLP43 and other members of the MLP family.

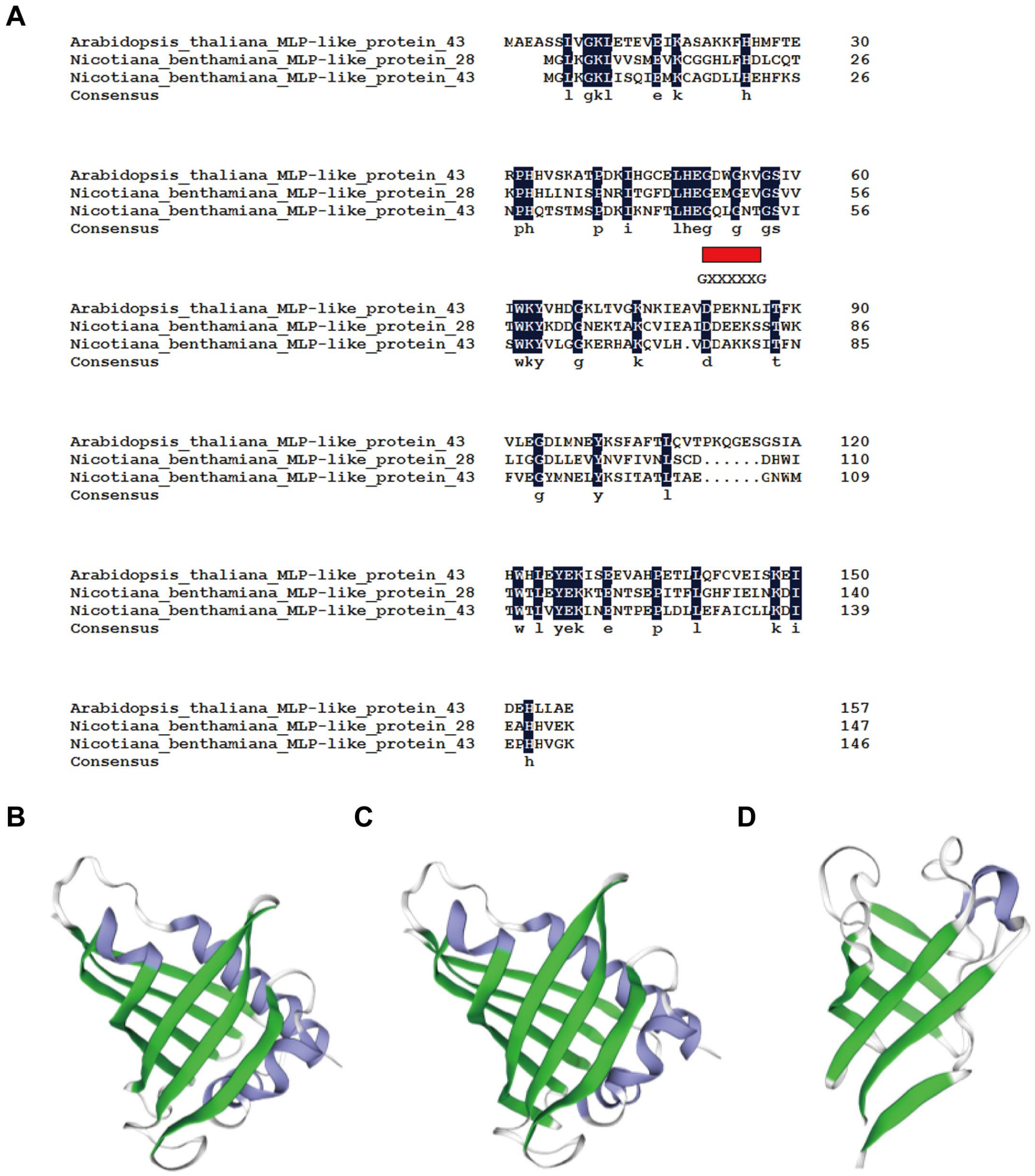

Fig. S3 Sequence analysis of NbMLP43. (A) Sequence Alignment of NbMLP43 with *Arabidopsis thaliana* MLP43 (NM_105756.4) and NbMLP28 (QEH62705.1) of *N. benthamiana*. (B-D) 3D-structure of NbMLP43 with *Arabidopsis thaliana* MLP43 and NbMLP28.

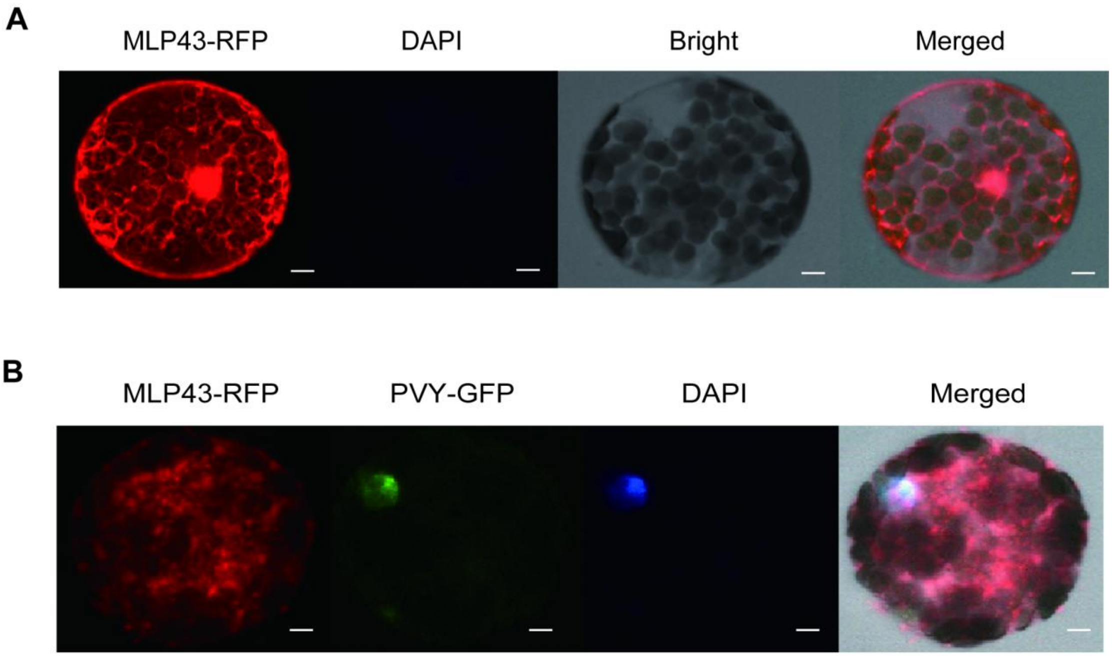

Fig. S4 The subcellular distribution of NbMLP43 in protoplasts. (A) Subcellular distribution of NbMLP43 observed in healthy *N. benthamiana*. (B) Subcellular distribution of NbMLP43 after PVY infection observed in protoplasts.

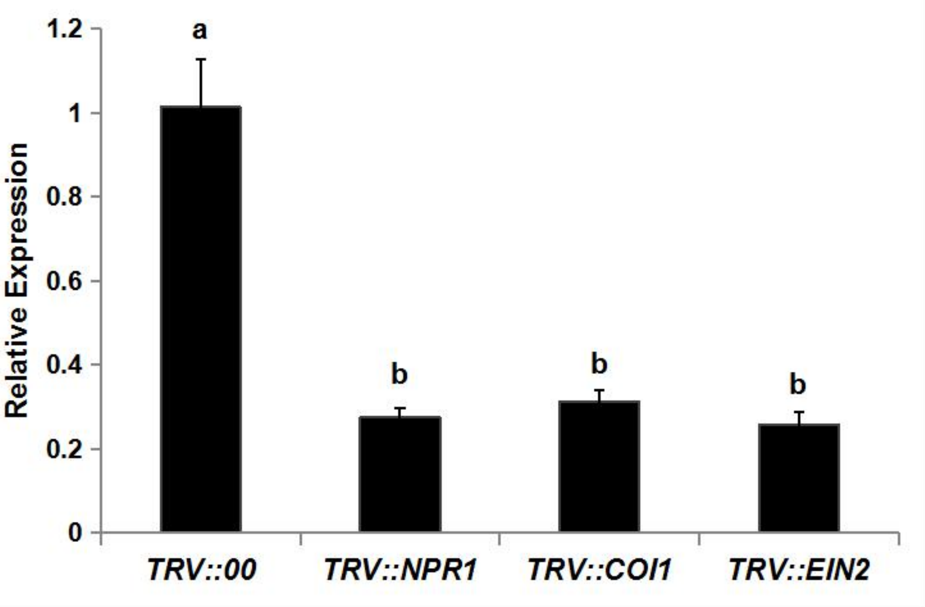

Fig. S5 Silencing efficiency of key signaling genes *NPR1*, *COI1* and *EIN2* in the signaling pathway.

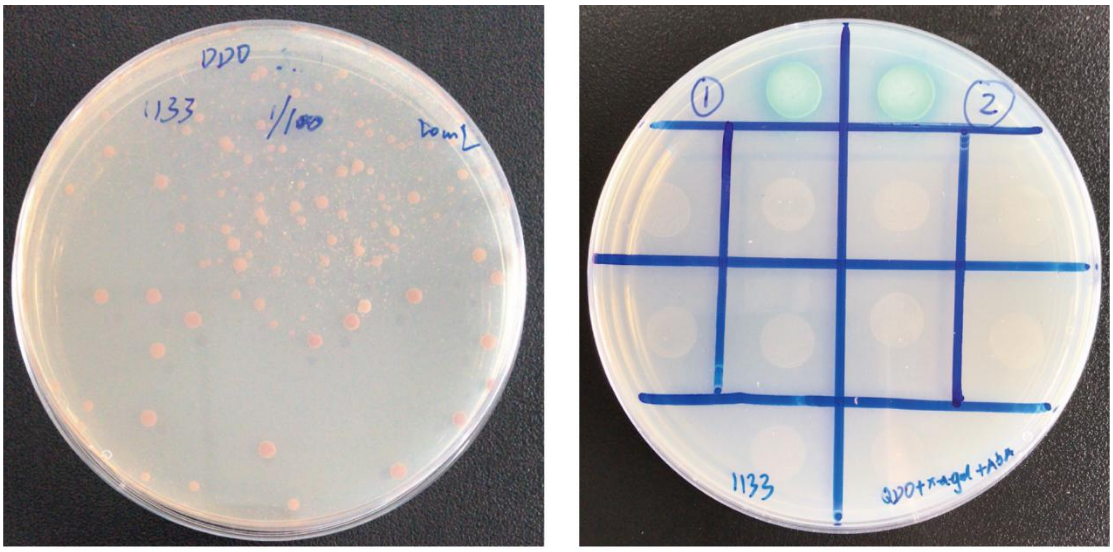

DDO QDO / X / A

Fig. S6 Screening of interactive proteins for NbMLP43 in yeast two-hybrid assay.

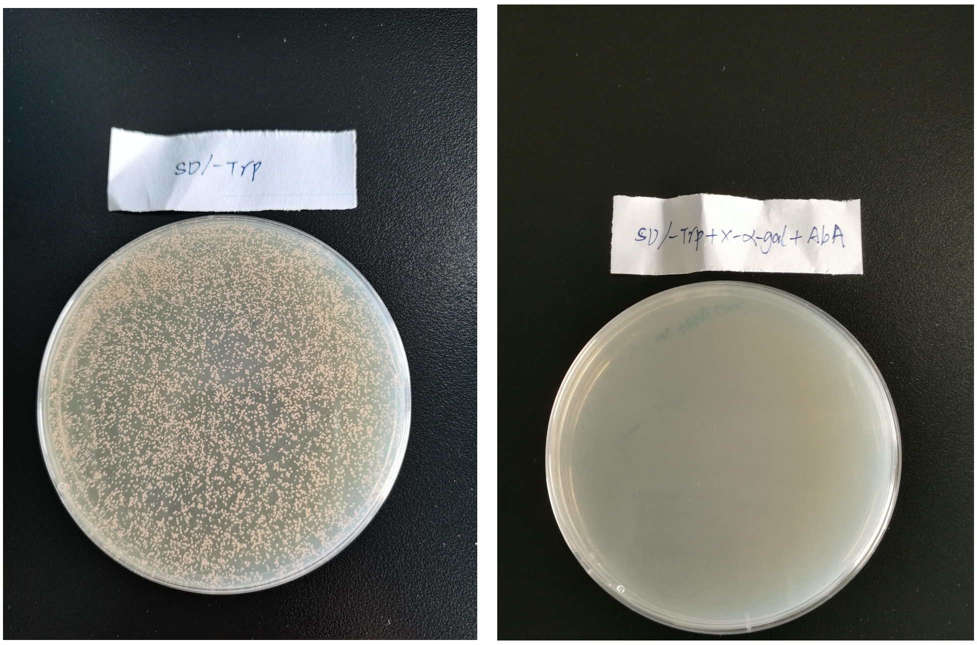

SD/-Trp **SD/-Trp / X / A**

Fig. S7 Self-activation verification test of BD-NbBBX24.
