# Supplementary material for: NbMLP43 Ubiquitination and Proteasomal Degradation via the Light Responsive Factor NbBBX24 to Promote Viral Infection
Source: Cells. 2023 Feb 11;12(4):590. doi: 10.3390/cells12040590 (PMC9954743; doi:10.3390/cells12040590)
Supplement: Supplementary file 1 [file cells-12-00590-s001.zip › Supplemental table S1.pdf]

Table S1 Primers used in this study

Primers	The sequence of primers
MLP43F	5’-ATGGGTCTCAAAGGCAAGTTGATCTC-3’
MLP43R	5’-CTATTTCCCGACATGATGAGGCTC-3’
MLP43 proF	5’- TCTCGGAACTCCAATTAGGATCCAATAAC-3’
MLP43 proR	5’-TTGTTAGCAGTGTAGATTTTCTT-3’
MLP43 QF	5’- CAAATCAAATCCACACCAAACC-3’
MLP43 QR	5’- CATGCCTCTCTTTTCCTCCAA-3’
β-Actin QF	5’-CAAGGAAATCACCGCTTTGG-3’
β-Actin QR	5’-AAGGGATGCGAGGATGGA-3’
NPR1-TRVF	5’-TAAGGTTACCGAATTCATGGATAATAGTGGGACTGCGT -3’
NPR1-TRVR	5’-AGACGCGTGAGCTCGGTACCCGGACTCCTCGCCGACAA-3’
COI1-TRVF	5’-TAAGGTTACCGAATTCCTTGATAATGGTGTCCGTGC-3’
COI1-TRVR	5’-AGACGCGTGAGCTCGGTACCCCAACGTATCCCAGAAGCATC-3’
EIN2-TRVF	5’-TAAGGTTACCGAATTCATGGAATCTGAAACTCTGACTATAG-3’
EIN2-TRVR	5’-AGACGCGTGAGCTCGGTACCATATTCTTCACTGCAAAT CTGGGC-3’
NPR1 QF	5’-ATCTCTTGCTATGGCAGGCGATG -3’
NPR1 QR	5’-ACCGTTGTCCTCTGTGCGTTG -3’
COI1 QF	5’-GCTCCACGCGATTACCAACGG -3’
COI1 QR	5’-CTGCCACCATCTCTTGCACACC -3’
EIN2 QF	5’-GTATGGAATTCAGGAGCGGAAGGC -3’
EIN2 QR	5’-AGAAGACGGAAGCACAAGAGCAAC -3’
pRGEB32-7s	5’-AAGCATCAGATGggcaAACAAAGCACCAGTGGTCTAG -3’
T1as	5’- CACCAAACCTCCACCATGTCTGCACCAGCCGGGAAT -3’
T1s	5’-GACATGGTGGAGGTTTGGTGGTTTTAGAGCTAGAAATA -3’
T2as	5’-AACTGACCCTCATGTAACGTTGCACCAGCCGGGAAT -3’

Primers	The sequence of primers
inf-T2as	5’-TTCTAGCTCTAAAACAACTGACCCTCATGTAACGT -3’
inf pRGEB32-7s	5’-AAGCATCAGATGGGCAAACAAA -3’
U6-7S	5’-TGTGCCACTCCAAAGACATCAG -3’
CRISPR-MLP43-dF2	5’-CGTGTAAGAAAATCTCACACTGC-3’
CRISPR-MLP43- dR2	5’-CACTTGTACCAAAGCAAACTCG-3’
MLP43-35SF	5’-CTTTAGATCTTCTAGAATGGGTCTCAAAGGCAAGTTG-3’
MLP43-35SR	5’-AGGAGGCCATGAATTCTTTCCCGACATGATGAGGCTC-3’
E100F	5’-CATCATTGCGATAAAGGAAAGGC -3’
E100R	5’-GGCGGTAAGGATCTGAGCTACAC -3’
PVY-F	5’-GATTTGCCTAAGGGTTGGTTTCG -3’
PVY-R	5’- GATGAATGGGCTTATGGTTTGGTG-3’
TMV-F	5’-GAGTAGACGACGCAACGG -3’
TMV-R	5’-CCAGAGGTCCAAACCAAAC -3’
CMV-F	5’-GTGGGTGACAGTTCGTAAA -3’
CMV-R	5’-GTGGGAATGCGTTGGT -3’
